# Targeting Sialic Acid Dependent and Independent Pathways of Invasion in *Plasmodium falciparum*

**DOI:** 10.1371/journal.pone.0030251

**Published:** 2012-01-12

**Authors:** Rosalynn Louise Ord, Marilis Rodriguez, Tsutomu Yamasaki, Satoru Takeo, Takafumi Tsuboi, Cheryl A. Lobo

**Affiliations:** 1 Department of Blood-Borne Parasites, New York Blood Center, New York, New York, United States of America; 2 Cell-Free Science and Technology Research Center, Ehime University, Matsuyama, Ehime, Japan; 3 Venture Business Laboratory, Ehime University, Matsuyama, Ehime, Japan; 4 Ehime Proteo-Medicine Research Center, Ehime University, Toon, Ehime, Japan; Weill Cornell Medical College, United States of America

## Abstract

The pathology of malaria is a consequence of the parasitaemia which develops through the cyclical asexual replication of parasites in a patient's red blood cells. Multiple parasite ligand-erythrocyte receptor interactions must occur for successful *Plasmodium* invasion of the human red cell. Two major malaria ligand families have been implicated in these variable ligand-receptor interactions used by *Plasmodium falciparum* to invade human red cells: the micronemal proteins from the Erythrocyte Binding Ligands (EBL) family and the rhoptry proteins from the Reticulocyte binding Homolog (PfRH*)* family. Ligands from the EBL family largely govern the sialic acid (SA) dependent pathways of invasion and the RH family ligands (except for RH1) mediate SA independent invasion. In an attempt to dissect out the invasion inhibitory effects of antibodies against ligands from both pathways, we have used EBA-175 and RH5 as model members of each pathway. Mice were immunized with either region II of EBA-175 produced in *Pichia pastoris* or full-length RH5 produced by the wheat germ cell-free system, or a combination of the two antigens to look for synergistic inhibitory effects of the induced antibodies. Sera obtained from these immunizations were tested for native antigen recognition and for efficacy in invasion inhibition assays. Results obtained show promise for the potential use of such hybrid vaccines to induce antibodies that can block multiple parasite ligand-red cell receptor interactions and thus inhibit parasite invasion.

## Introduction

In *Plasmodium falciparum*, the causative agent of the most lethal form of human malaria, the cyclical bursting and invasion of erythrocytes is responsible for all the clinical manifestations of the disease [1]. Continued survival of the parasite in the human host requires successful invasion of merozoites into uninfected erythrocytes. This is an active and sophisticated process, and requires multiple steps of interaction among receptors on the red cell and parasite ligands [2]. *P. falciparum* has developed the ability to invade red cells using multiple parasite ligand-erythrocyte receptor interactions that have become known as alternative invasion pathways [3]. Various parasite proteins can fulfill similar roles in the invasion process and hence any successful malaria vaccine will have to target all alternative pathways of invasion.

Two major types of invasion pathways have been described in *P. falciparum*: a sialic acid (SA) dependent pathway and a SA independent pathway. Two families of parasite ligands have been implicated in these invasion pathways. Proteins of the Erythrocyte Binding Ligands (EBL) family are stored in the micronemes, after production in the endoplasmic reticulum, and include EBA-175 [4], EBA-140 [5], [6], EBA-181 [7], [8] and EBL-1 [9]. Glycophorins (GP) A [10], B [11], [12] and C [13] have been identified as the receptors to which EBA-175, EBL-1 and EBA-140 bind, respectively. The receptor to which EBA-181 binds has yet to be identified, but has been found to be neuraminidase and chymotrypsin sensitive, and trypsin resistant [7]. As the glycophorins are the major sialylated proteins on the erythrocyte, these parasite proteins largely govern the sialic acid (SA) dependent pathways of invasion.

In contrast, the second family of parasite ligands that mediate invasion are the *P. falciparum* reticulocyte binding protein-like homologues (PfRHs), PfRH1, PfRH2a, PfRH2b, PfRH3, PfRH4, and PfRH5, and these act largely through SA independent pathways [14]–[16]. RH1 is an exception in this group as it has been found to interact with erythrocytes in a sialic acid dependent manner [17]. The erythrocyte receptors for these proteins remain unknown, except for RH4, which has been found to adhere to complement receptor 1 (CR1) [18].

During the process of invasion, merozoites are unprotected within the blood stream. They are exposed to circulating host immune factors and, in natural human populations, encounter a heterogeneous population of erythrocyte surface proteins. Whilst there is evidence that antibodies against native EBL and RH proteins can inhibit invasion [19], it has also been shown, however, that there is differential expression of these proteins [20]–[22]. This results in phenotypic variation of the invasion profiles. Such variation provides the parasite with the ability to evade antibody-mediated immune mechanisms and to utilize those erythrocyte surface proteins immediately present in the host blood cells. It is unlikely that vaccines incorporating only a single blood-stage antigen will be sufficient to provide adequate protection against the severity of the disease seen in malaria endemic areas given the extensive diversity of *Plasmodium*'s invasion repertoire and the variability of the human immune response.

In an attempt to dissect out the invasion inhibitory effects of antibodies against ligands from both the SA dependent and SA independent pathways, we have used EBA-175 and RH5 as model members of each pathway. EBA-175 binds to GPA for invasion of erythrocytes, requiring both the sialic acid residues as well as the peptide backbone of GPA for successful binding [10]. EBA-175 and other EBA family genes contain six extracellular regions, of which only region II binds erythrocyte receptors [19], [23]. Despite the expression of this ligand by all *P. falciparum* clones, the effect of antibodies to region II of EBA-175 on the invasion of erythrocytes is variable [19], [24]–[26]. EBA-175 is used by a wide variety of parasite clones for invasion, and a recent paper [27] shows that antibodies specific for EBA-175 block erythrocyte invasion through the EBA-175/GPA pathway. The authors also reported that inhibition of parasite invasion by antibodies to region II of EBA-175 is not affected by polymorphisms occurring in region II. RH5, while being the smallest member of the PfRH family at 65kDa, appears to have a critical role in invasion as attempts to disrupt the gene have not been successful [2], [28]. Unlike other genes encoding merozoite surface proteins, such as *Pfama1*, there have been few non-synonymous mutations observed so far in the gene encoding PfRH5 [28], and there have been no reports to date of any genetic diversity studies from natural parasite populations. In this study, we analyzed the individual and combined effects of antibodies against these two parasite ligands on invasion. Results presented in this study validate the use of a combination of these two ligands as a potential vaccine that would have broad activity against *P. falciparum*.

## Results

### Antigen expression and testing for function

**Figure 1:**
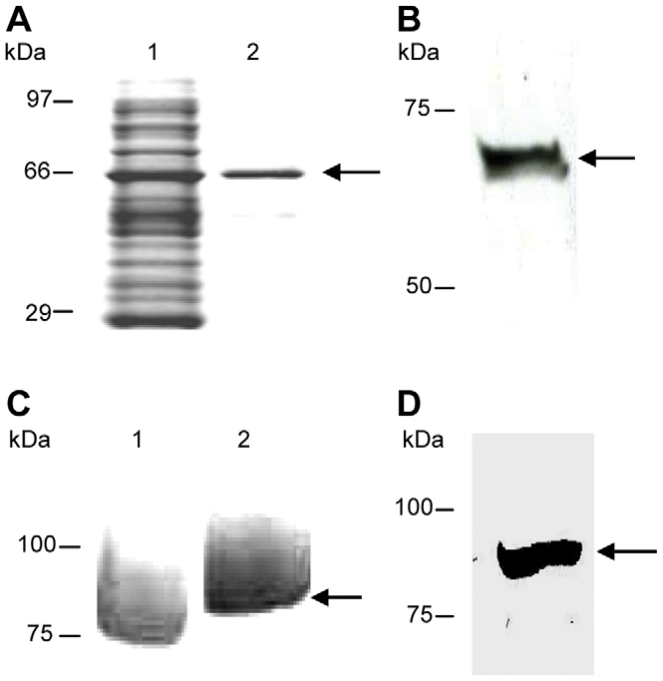
Recombinant EBA-175 and RH5 antigens are stable, pure and expressed in the correct conformation. The non-reduced (Lane 1 of panel **A**) and the reduced elution (Lane 2 of panel **A**; both visualized by Coomassie staining) of region II of EBA-175 synthesized using the yeast expression system *Pichia pastoris*, and the binding of this recombinant to normal erythrocytes (panel **B**), confirm correct expression and conformation of the EBA-175_RII_ antigen with the expected product of ∼80 kDa (indicated by the arrows in Lane 2 of panel **A** and panel **B**). The elution of full length RH5 synthesized using the wheat-germ synthesis (panel **C**, indicated by the arrow), and binding of rRH5 (panel **D**) to normal erythrocytes indicates functional conformity of this recombinant antigen, as shown by the presence of a single product at the expected size of ∼63 kDa (indicated by arrows in both **C** and **D**).

The members of the PfRH protein family have no obvious domain structures, such as the region II cysteine-rich domains of the EBL family. We therefore decided to use the entire PfRH5 protein as the immunogen as additionally, differing reports of ability to inhibit parasite invasion were reported based on the region of PfRH5 used for immunization [2], [16], [29]. It was also important to determine that the recombinant proteins used for immunization were conformationally similar to the native parasite proteins. Thus, besides looking at the purity and stability of the products, we also assessed the binding ability of these recombinant proteins to erythrocytes. We chose to express RH5 in the wheat germ system on account of the multiple advantages afforded by it. It is a eukaryotic expression system that has no glycosylation machinery, thus mimicking native *Plasmodium* antigen expression. It can be established as a high-throughput platform (in 6 well robotic formats), resulting in total yields of ∼2 mg protein overnight. There has been a high success rate of expression reported in literature [30], [31] from genome-wide malaria, human, and plant projects. The successful expression of the full-length recombinant RH5 (hereafter rRH5), produced by the wheat-germ synthesis method was confirmed by the presence of a ∼63 kDa product on SDS-PAGE gel after elution of the total protein preparation, as shown in Figure 1A, lane 1. This rRH5 antigen also binds to normal erythrocytes: this was determined by loading the eluate from the Ni column purification onto an SDS-PAGE gel, the presence of where the expected a ∼63 kDa was visible (see Figures 1A, lane 2 and 1B). This confirms that this method of expression was effective at producing a functionally conformed antigen. As we propose that rRH5 would be a candidate to add to the current EBA-175 vaccine regime, we have used the same sub-fragment of the EBA-175 antigen encompassing region II (EBA_175_RII_) that is being utilized in clinical trials (Figure 1C). Thus, although correct expression of this recombinant has been determined elsewhere [32], we also have independently determined its conformity by erythrocyte binding, resulting in the expected ∼80 kDa product, as shown in Figure 1D.

### Anti-EBA-175_RII_ and anti-rRH5 antibodies recognize native parasite protein

**Figure 2:**
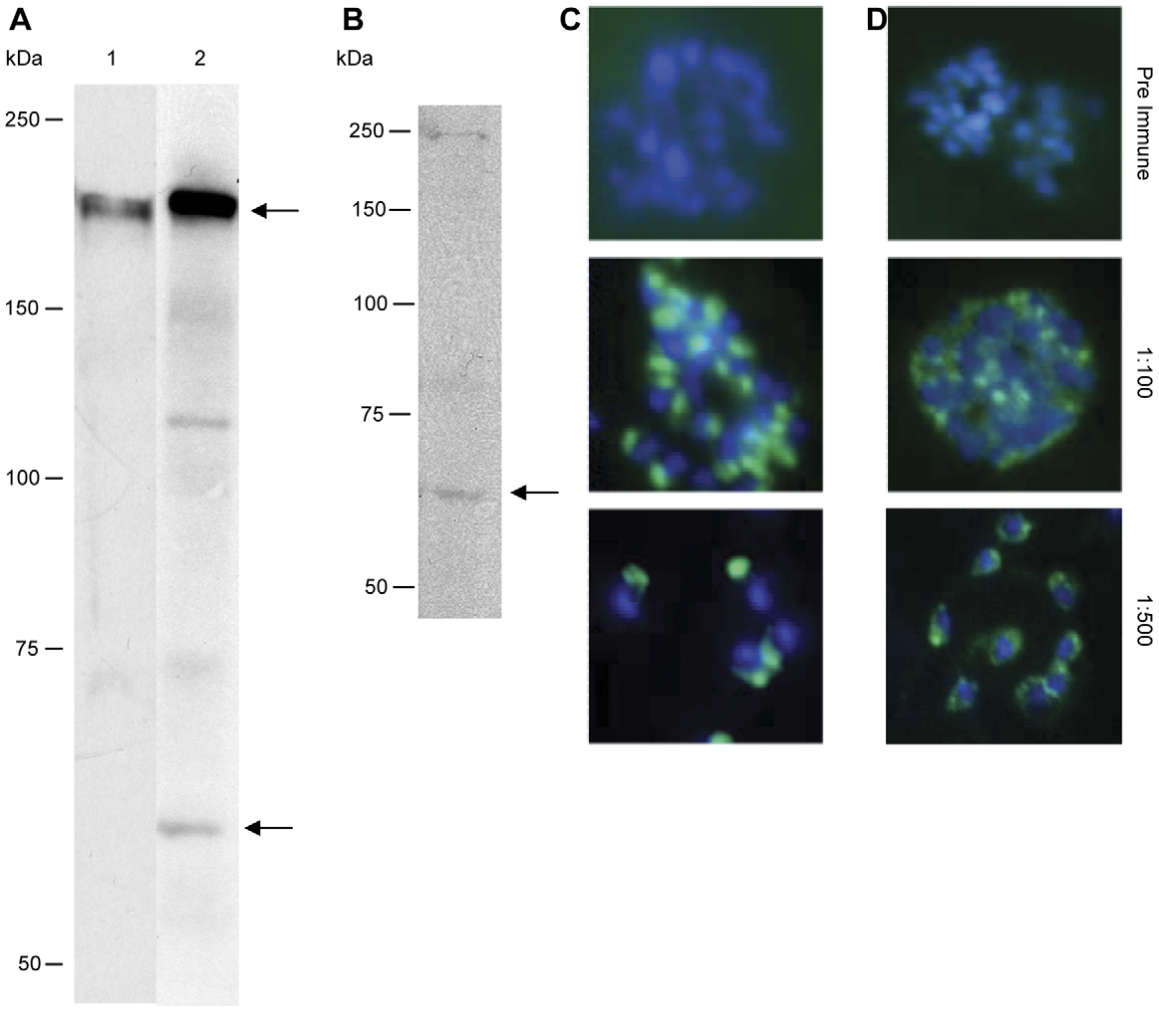
Recognition of native proteins. Anti-EBA-175_RII_ antibodies are able to detect native EBA-175 protein of 175 kDa from 3D7 parasite lysates (Lane 1, panel **A**). Anti-EBA-175_RII_/rRH5 (sera from the combined vaccine) is able to detect both EBA-175 and RH5 native proteins from the same lysate (Lane 2, panel **A**; EBA-175 indicated by top arrow; RH5 indicated by bottom arrow). Anti-rRH5 antibodies are specific for the native 65 kDa RH5 protein from 3D7 parasite lysate (panel **B**; indicated by arrow). All products were visualized with ECL after immunoblotting. Immunofluorescent detection of EBA-175 and RH5 in mature and rupturing 3D7 schizonts. Parasites were labelled with anti-EBA-175_RII_ (panel **C**) or with anti-rRH5 (panel **D**) at dilutions of 1∶100 and 1∶500, then the slides were incubated with FITC-conjugated anti-mouse IgG (green) and mounted with 10 mg/mL DAPI (blue). EBA-175 and RH5 are each localised to the apical end of merozoites. Non-staining with preimmune sera (1∶100 dilution) confirm specificity of each antibody to its respective antigen.

Mice were immunized with full-length rRH5, or rEBA-175_RII_, or a combination of both, and ascites and cardiac bleed sera were obtained at the end of the immunization regimen. It is important to ensure that the sera produced recognize and react with the specific native parasite proteins to ensure any effect mediated by antibodies contained therein is specific to the immunogen. Therefore, all sera produced were assayed on both Western Blots of parasite extracts as well as in IFAs on smears of mature stage parasites. Immunoblotting with sera obtained on native parasite lysate (3D7) showed the anti-EBA-175_RII_ antibody recognized native EBA-175, as determined by the 175 kDa product on SDS-PAGE gel (Figure 2A, lane 1). Immunoblotting with antibodies against the rRH5 protein showed that these antibodies are also specific for the native RH5 protein (Figure 2B). When sera from mice immunized with the combination of the two antigens, both a 175 kDa product and the native RH5 protein of 65 kDa are obtained (see Figure 2A, lane 2). The specificity of anti-EBA-175_RII_ and anti-rRH5 to native protein was independently confirmed by immunofluorescence, using FITC-conjugated anti-mouse IgG and DAPI staining, and further confirmed that native EBA-175 and RH5 are localized to the apical end of merozoites, as expected for parasite proteins involved in invasion (see Figures 2C–2D).

### Confirmation that anti-EBA-175_RII_ is effective at inhibiting invasion of the parasite 3D7 isolate

**Figure 3:**
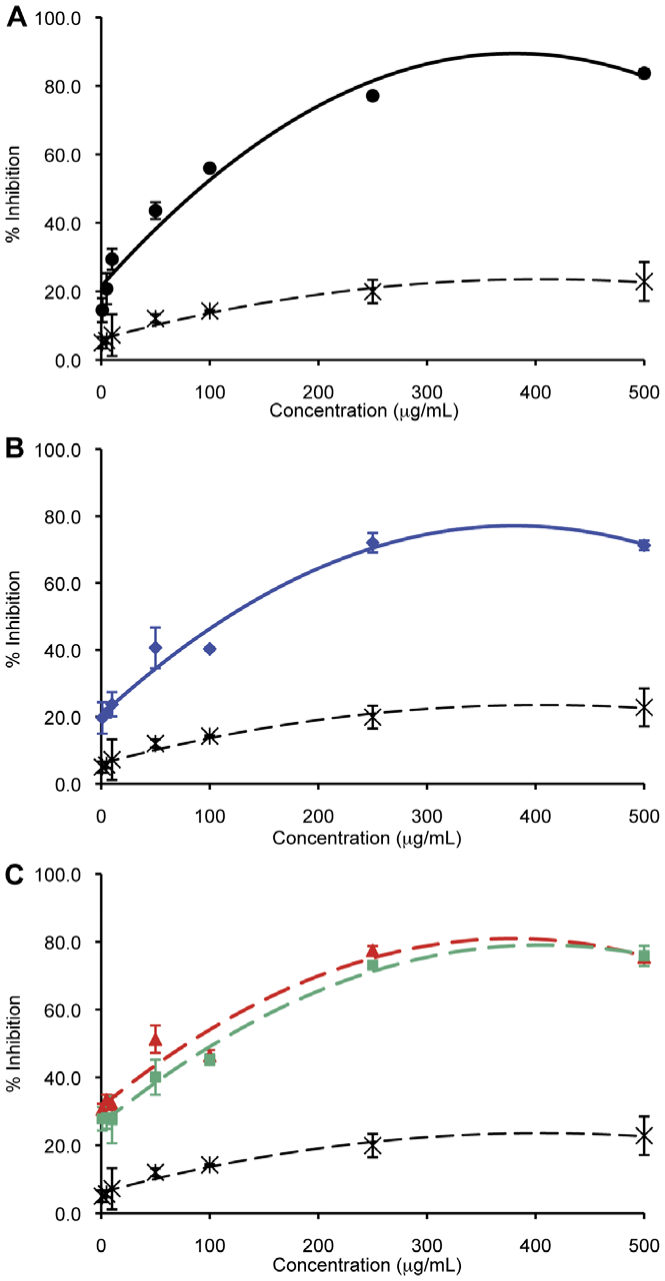
Invasion Inhibition of 3D7 with anti-EBA-175_RII_ and anti-rRH5 antibodies. Anti-EBA-175_RII_ (solid black line in panel **A**) and anti-rRH5 antibodies (solid blue line in panel **B**) inhibit invasion of 3D7 in a linear correlation to a similar extent. The two varying combinations used, anti-EBA-175_RII_/anti-rRH5 and anti-EBA-175_RII_+anti-rRH5 (solid red and solid green lines, respectively, in **C**), also showed the same positive correlation between increased antibody concentration and % inhibition (the color key for each antibody is conserved from **A**, **B**, and **C**). Percentage invasion inhibition from purified mouse IgG used as a control is shown as dashed black line (**A**, **B**, **C**).

After purifying the IgG fraction from the different sera, invasion inhibition assays (IIAs) in 250 µL cultures were established with total antibody concentrations ranging from 1.0 µg/mL to 500 µg/mL. All cultures were initiated at 0.8% parasitaemia using mature parasites (schizonts) purified off a Percoll gradient, and the resulting parasitaemia after 24–30 hours (i.e. after only one round of invasion) was determined. A dose-dependent curve of inhibition was obtained using IgG from EBA-175_RII_ immunized mice and the degree of inhibition increased from 15%, at 1 µg/mL, to 84%, 500 µg/mL, compared to the no IgG control (Figure 3A). Inhibition assays performed with control IgG in this same range of concentrations yielded 5.0% to 23% inhibition. This confirms the specificity of the inhibition. However we observe a plateauing effect at 250 µg/mL, when the effective inhibition reaches 77% inhibition (see Figure 3A) and concentrations higher than this do not yield significantly higher inhibition. Previous studies with antibodies produced against the same EBA-175_RII_ antigen in rabbits have shown up to ∼80% growth inhibition [32], [33], suggesting that this antigen is an effective immunogen, and provides encouragement for continuing with this antigen in human studies. Of note however, when this same EBA-175_RII_ antigen was used to immunize malaria-naïve adults, only a low level of growth inhibition was observed (10–20%) despite high antibody titers [34], suggesting the need for additional components in an invasion targeting vaccine.

### Anti-rRH5 antibodies exert potent anti-invasion effects in 3D7

This is the first study to test the effect of antibodies against full-length rRH5 on invasion of *P. falciparum*, and we obtained potent degrees of invasion inhibition ranging from 20% at 1.0 µg/mL, to a maximum of 72% at 250–500 µg/mL (Figure 3B). There is no difference in the levels of inhibition obtained from anti-rRH5 or anti-EBA-175_RII_ between 1 µg/mL and 50 µg/mL, although, at the higher concentrations (100 µg/mL to 500 µg/mL), the level of inhibition from anti-EBA-175_RII_ does exceed that of anti-rRH5 (P<0.05, T-test). These data are the first to indicate that PfRH5 alone is an effective immunogen, capable of eliciting an effective inhibitory immune response in these mice.

### Combination anti-EBA-175_RII_ and rRH5 antibodies show synergistic effects at the lower end of the concentration range

Apart from determining the inhibitory effects of antibodies to full-length RH5, the other purpose of this study was to look for synergistic effects on inhibition of invasion in the presence of antibodies to both EBA-175 and RH5, in order to block multiple ligands required for merozoite invasion simultaneously, thereby overcoming functional redundancy among invasion ligands and the capacity for immune evasion. Thus, IIAs were performed with two combination variants: anti-EBA-175_RII_/rRH5 (obtained from immunizing mice with a single vaccine containing equal quantities of each antigen) and an artificial in-tube combination of the two IgGs after purification. This second combination used equal quantities of purified IgG from each individual serum, with 50% of the total IgG concentration coming from each antibody. As expected, there is a linear increase in invasion inhibition directly correlated to the increase in antibody concentration for both types of combination, and there is no significant difference between the level of inhibition obtained from these two types of combinations at any concentration (P>0.05, T-test; Figure 3C).

**Figure 4:**
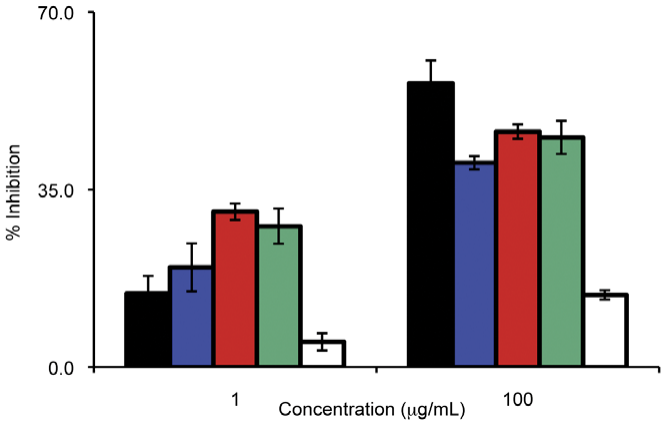
Anti-sera against the hybrid vaccine show synergistic effects at low concentrations. The invasion inhibition of both combination sera are greater than those obtained from the individual sera at the lowest concentrations used, 1 µg/mL. The combinations contain 50% of each individual immunogen (in the case of the combination vaccination, anti-EBA-175_RII_/rRH5) or sera (in the case of the in-tube combination, anti-EBA-175_RII_+anti-rRH5). However, by 100 µg/mL, the synergistic effects of the combinations are no longer apparent, and the inhibition from the combinations is equivalent to ∼50% contribution from the two individual sera (anti-EBA-175_RII_ shown by black bars, anti-rRH5 shown by blue bars, anti-EBA-175_RII_/rRH5 shown by red bars, anti-EBA-175_RII_+anti-rRH5 shown by green, control IgG shown by white bars).

Interestingly, we observe two patterns of inhibition at the different ends of the concentration spectrum. At the lower concentrations of antibody (1.0 µg/mL – 5.0 µg/mL) we obtain a synergistic effect with the combination IgG a as these antibodies exert a greater inhibition on invasion than antibodies from the single immunogen sera used at the same concentration: 31% and 28% inhibition for the anti-EBA-175_RII_/rRH5 and anti-EBA-175_RII_+anti-rRH5, respectively, compared to 15% and 20% inhibition from the single anti-EBA-175_RII_ and anti-rRH5 IgG, respectively (P<0.05, T-test for anti-EBA-175 *vs*. either combination; see Figure 4). As the concentration of antibody used in the IIA increases, however, this synergy is replaced by a different type of inhibitory profile where we obtain intermediate levels of inhibition to that observed for the single antibodies. Thus, at these concentrations (250 µg/mL to 500 µg/mL), the level of inhibition is equivalent to 50% contribution from anti-EBA-175_RII_ plus 50% from anti-rRH5, suggesting that the individual components are acting independently to each other. At 100 µg/mL, the level of inhibition from each combination is only as great as anti-EBA-175, indicating that around 100 µg/mL, there is a transition from a synergistic effect to an independent effect. It is possible that when both antibodies are present and in excess, any synergistic effect is masked by some steric hindrance. If so, this would mean it is possible that as an individual antibody binds to its specific antigen, it creates a physical block for the other antibody to reach its antigen, thus the antigen is still available for binding to its erythrocyte receptor for invasion. We are currently investigating underlying mechanisms that could form the basis of this transition. Of interest to vaccine design and deployment, current studies indicate that titers of antibodies in vaccinated people would approximate the lower end of the concentration spectrum [34] and thus synergistic effects of antibodies from such combination vaccines would be expected to be obtained.

### Inhibition of 3D7 parasite invasion in neuraminidase-treated erythrocytes confirms SA dependence of EBA-175 and SA independence of RH5

**Figure 5:**
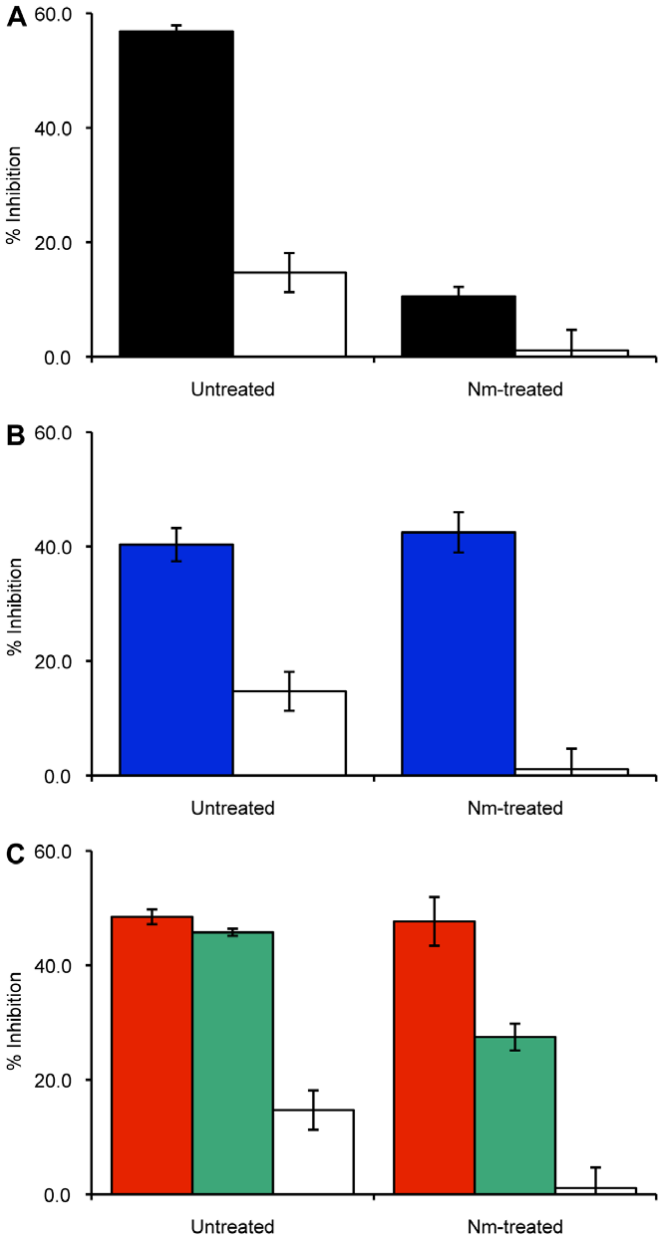
Enzymatic (neuraminidase) treatment of erythrocytes eliminates the inhibitory effects of anti-EBA-175_RII_ antibodies but has only a mild effect on anti-rRH5 sera. Invasion inhibition assays with 3D7 and neuraminidase-treated cells (100 µg/mL antibody used) show that any inhibition due to the presence of anti-EBA-175_RII_ antibodies is masked by the removal of ligands with sialic acid compared to untreated cells (**A**; anti-EBA-175_RII_ and IgG shown as solid black and solid white bars, respectively). Anti-rRH5 alone (**B**; solid blue bars), or in combination (**C**; anti-EBA-175_RII_/rRH5 and anti-EBA-175_RII_+anti-rRH5 shown as solid red and solid green bars, respectively) is still able to significantly inhibit growth in treated cells as a sialic acid independent pathway is utilized by the RH5 antigen.

Treatment of erythrocytes with enzymes that selectively cleave moieties of malarial receptors would result in elimination of the use of that specific invasion pathway. We thus decided on the use of neuraminidase (Nm) that removes sialic acid residues from red cell receptors, such as GPA, the EBA-175 receptor. We reasoned this would eliminate the EBA-175 contribution to the invasion arsenal as its cognate receptor was impaired. Our hypothesis was confirmed when the invasion inhibition that was obtained in untreated erythrocytes in the presence of anti-EBA-175_RII_ IgG effectively disappeared when Nm-treated cells were used in the invasion assay (Figure 5A). PfRH5, on the other hand, is known to bind to a non-sialylated receptor [2], [16], [28], [29] and thus, as expected, we obtained a minimal difference in the invasion inhibition effect from anti-rRH5 between untreated and Nm-treated erythrocytes (Figure 5B). When the antigen combinations are used with Nm-treated cells, the expectation is that any invasion inhibition observed could only be from the action of the anti-rRH5 portion of the antibodies (the anti-EBA-175_RII_ portion being blocked by the enzymatic treatment), and this is observed in the second combination when the independent antibodies are combined after purification: the invasion inhibition is reduced by ∼60% from 46% in untreated cells to 28% in Nm-treated cells (Figure 5C). These data confirm that RH5 utilizes a SA independent pathway for invasion, as previously shown [16], and when the EBA-175/GPA pathway is blocked, 3D7 parasites will utilize this alternative, SA independent, pathway for invasion. However, there is no significant decrease from the anti-EBA-175_RII_/rRH5 combination as expected (Figure 5C). This suggested that the RH5 portion of the combination vaccine was perhaps more immunogenic than the EBA-175_RII_ component in these mice. However, titrating the sera against recombinant EBA-175_RII_ and recombinant RH5 showed that both sera had approximately the same titers (∼1∶100,000, data not shown). Future work will be directed to understand this discrepancy between the two combinations.

### Alternative pathways are used by the parasite for invasion

**Figure 6:**
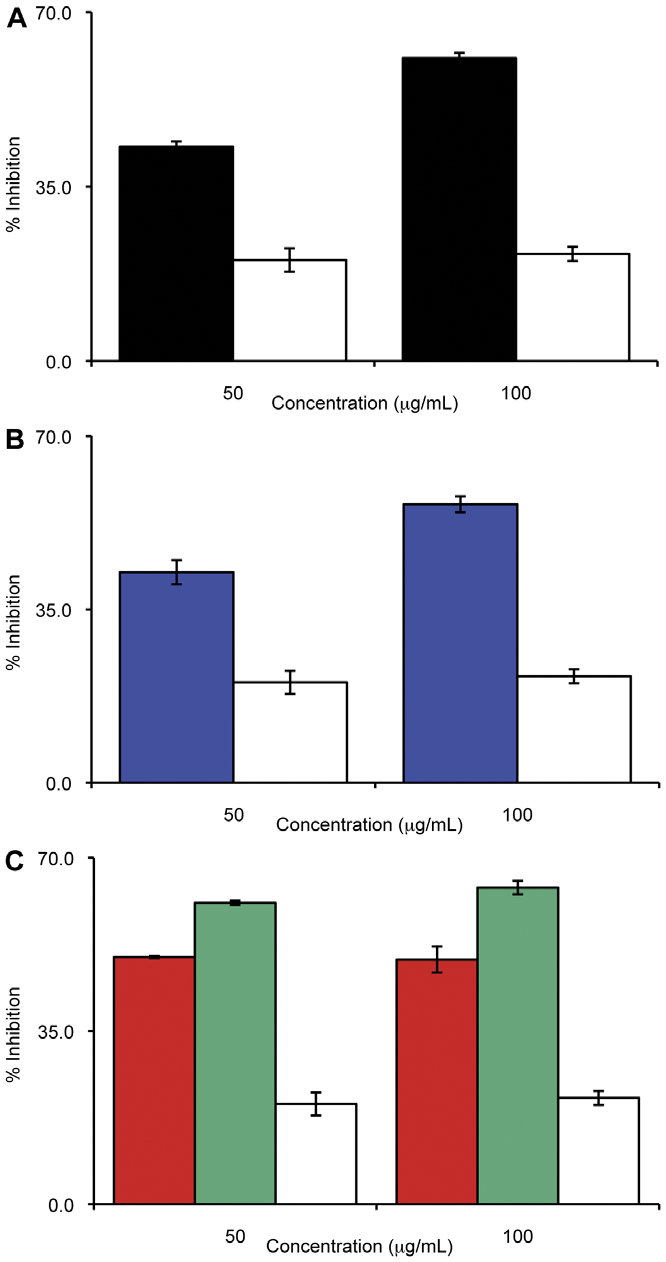
Dd2 parasites are not wholly dependent on the EBA-175/GPA pathway of invasion. In the presence of anti-EBA-175_RII_ antibodies (**A**; black bars), inhibition when using the Dd2 strain is only 60% (Ab concentration of100 µg/mL), compared to 55% inhibition from 3D7, suggesting alternative SA dependent pathways are utilized by Dd2, such as EBL-1/GPB or EBA-140/GPC (for **A**, **B** and **C**, Dd2 with its control IgG shown as solid white bars). Although anti-rRH5 antibodies (**B**; blue bars), should only block a SA independent pathway, there is still greater inhibition with Dd2 compared to 3D7. In the presence of both antibodies (**C**; anti-EBA-175_RII_/rRH5 shown as red bars and anti-EBA-175_RII_+anti-rRH5 shown as green bars, respectively), there is a significant difference in the ability of Dd2 to invade compared to 3D7, especially when individual antibodies are combined.

Different parasite strains use different erythrocyte receptors for invasion, such that some strains are totally dependent upon a single pathway while others may have the ability to avail of multiple routes of invasion. It will be necessary that a malaria vaccine target all these different parasite strains. We thus decided to run the IIAs with the same set of sera on a parasite strain that has a very different invasion profile from 3D7 (SA independent). We chose Dd2 as it has been shown to be totally dependent upon GPA for invasion such that Nm-treatment of erythrocytes results in 100% inhibition of invasion in this strain [20], and the EBA-175/GPA pathway has been presumed to be the dominant SA dependent route of entry in this strain. Thus, we expected a high inhibition of invasion with Dd2 in untreated erythrocytes when anti-EBA-175_RII_ antibodies are present: these antibodies will block the native EBA-175 antigen, preventing the EBA-175/GPA pathway required for invasion, mimicking the effect of Nm-treatment on erythrocytes. However, we observed no more than ∼60% inhibition of invasion (at 100 µg/mL; Figure 6A). Although this is significantly greater than the inhibition seen with 3D7 at the same antibody concentration (P = 0.015, T-test), this is not a complete block as seen when Dd2 invasion is assayed in Nm-treated cells [35], [36]. These results suggest that, contrary to published reports, alternative SA dependent pathways (i.e.EBL-1/GPB, EBA-140/GPC) are being utilized in Dd2 when the EBA-175/GPA pathway is not available. Further investigation using alternative SA dependent strains, such as W2-mef, are needed to elucidate this apparent paradigm. The inhibition profile when anti-rRH5 antibodies were used in the invasion assay are similar to those obtained with anti-EBA-175_RII_ in that the level of inhibition at 50 µg/mL was equivalent to that of 3D7 (43% in Dd2, compared to 41% in 3D7; see Figure 6B). However, at 100 µg/mL, the inhibition observed was significantly higher in Dd2 than 3D7 (57% compared to 40%, respectively; P = 0.003, T-test). Interestingly, there was no difference in level of inhibition between the two strains for the anti-EBA-175_RII_/rRH5 combination (antibodies from a combined vaccination) at either concentration (Figure 6C) but the level of inhibition observed in the presence of the anti-EBA-175_RII_+anti-rRH5 (antibodies combined after purification) was significantly higher in Dd2 at both concentrations (P<0.02, T-test).

## Discussion

To control and eventually eradicate malaria, an effective vaccine is considered to be necessary, in addition to currently existing tools, such as drugs and insecticide treated nets. The main requirement for the development of a successful anti-invasion malaria vaccine is the demonstration that antibodies made against each ligand can block the erythrocyte invasion of parasites. An *in vitro* parasite invasion inhibition assay (IIA) is one of the widely-used assays that can measure the functional activity of antibodies against asexual erythrocyte stages of *Plasmodium*. While it is still controversial whether the inhibitory activity measured by the IIAs reflects protective immunity induced by a malaria vaccine, the assay has been used in multiple preclinical and clinical studies as a prime immunological readout [27], [37] and we have used it in this study to look at relative efficacy of two invasion ligands alone and in combination to elicit inhibitory antibodies.

EBA-175, specifically region II of this antigen, is currently a leading anti-malarial vaccine candidate, and EBA-175_RII_ has been shown to be safe and immunogenic in malaria-naïve adults [34], [38]. Further, the anti-sera from these vaccinated individuals inhibit, but do not eliminate, the growth *P. falciparum*, suggesting that additional components to the vaccine may be needed. As *P. falciparum* has been shown to invade erythrocytes by multiple pathways, using both the EBL and PfRH families a vaccine that is able to target more than one pathway is desirable. Recent studies have shown that the members of the EBL and PfRH families act cooperatively in merozoite invasion [37] and this has important implications for vaccine development. We chose to test EBA-175 in combination with PfRH5 as researchers have so far been unable to produce a PfRH5-knock-out, suggesting that this antigen is essential for parasite invasion. Previous studies with RH5 report a mixed success with inducing invasion inhibitory antibodies that could be ascribed to the use of different regions of RH5 as immunogen [16], [28], [29]. So far, no studies have been done with full-length RH5 and to ensure a thorough validation of this ligand as a vaccine candidate, we undertook to assess the functionality and immunogenicity of the full-length RH5 protein alone and in conjunction with EBA-175_RII_.

The rationale for including EBA-175_RII_ in a multi-component vaccine has been well established by previous studies and these have shown that not only does this antigen elicit a suitable immune response, but anti-EBA-175_RII_ antibodies are able to inhibit invasion of merozoites into erythrocytes by blocking the specific, SA dependent, EBA-175/GPA pathway [33] but by also inhibiting an alternative, SA independent, pathway [24], [39]. Further, both the DNA and recombinant protein forms of the EBA-175_RII_ vaccine component have been found to be safe and immunogenic in animal models and malaria-naïve human trials, respectively [25], [26], [34].

Our results with mouse anti-EBA-175_RII_ antibodies show similar invasion inhibition in both 3D7 and Dd2 parasite strains and confirm the vaccine potential of this malarial ligand. A surprising find was that anti-EBA-175_RII_ antibodies did not totally eliminate invasion of Dd2 as seen in its invasion of neuraminidase treated cells. The EBA-175/GPA pathway is thought to be the predominant route of entry for SA dependent parasite strains but this study shows that efficient invasion persists for this strain in the presence of potent anti-EBA-175 antibodies. Thus, other SA dependent pathways, EBL-1/GPB and EBA-140/GPC may be operating to overcome the loss of the GPA mediated pathway. Selection for these alternative ligand mediated pathways was not needed over time as was seen in the creation of the Dd2-Nm strain [35], [36]. Further molecular analysis of gene expression of parasites in these IIA assays may shed light on the identity of these parasite ligands.

This is the first study to characterize the antibodies generated to full length RH5 and we report that the invasion-inhibitory potential of these antibodies equals that generated by EBA-175_RII_ for both 3D7 and Dd2 parasites. Purified anti-rRH5 immunoglobulins recognized the native parasite ligand and inhibited merozoite invasion in a dose-dependent manner resulting in up to 72% inhibition at 500 µg/ml. These results indicate a possible reason for the inability to successfully delete the gene encoding RH5 from the parasite genome and stress the importance of determining the candidacy of the various EBL and RH ligands in such IIAs before designing rational vaccine combinations that will induce immune responses that ultimately may disrupt merozoite invasion.

**Figure 7:**
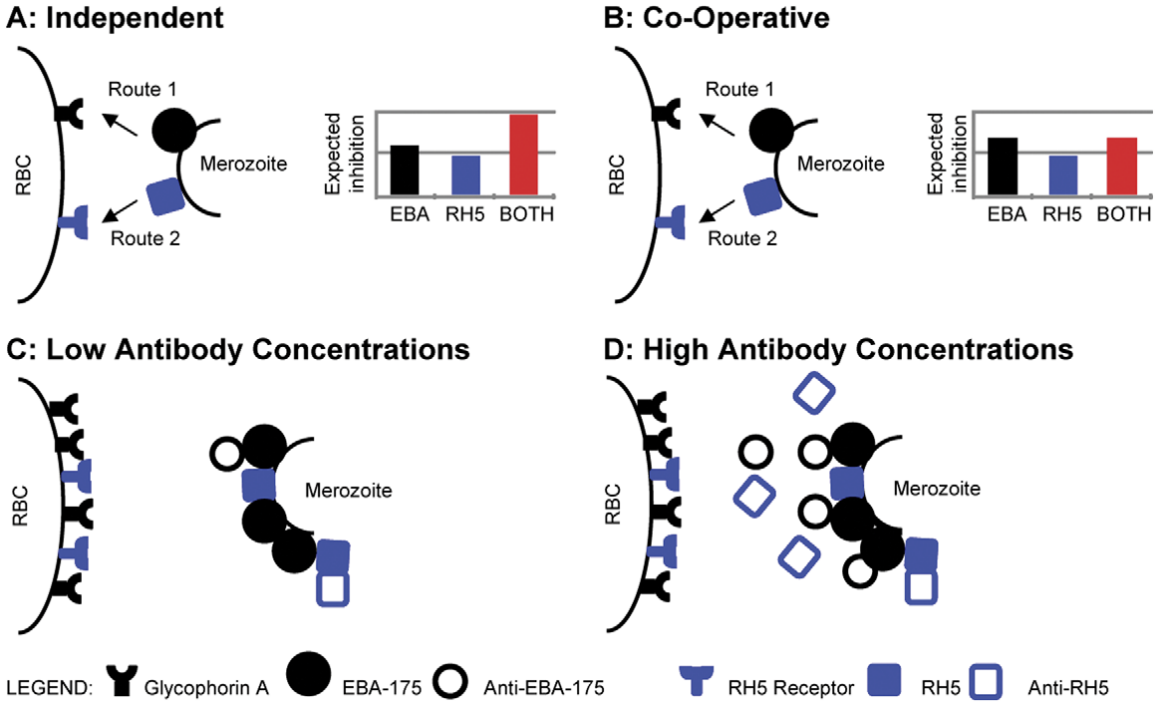
Independent Antibody Interaction and Co-Operative Antibody Interaction Models. ***Independent Antibody Interaction Model*** (Panel **A**): If the ligand-receptor interactions are independent of each other, invasion by either EBA-175 (Route 1) or by RH5 (Route 2) is not affected by the other. Thus, in the presence of both anti-EBA-175_RII_ and anti-rRH5 antibodies, the expected inhibition from the combination anti-sera is ADDITIVE (synergistic) when compared to the inhibition from the individual anti-sera. ***Co-Operative Antibody Interaction Model*** (Panel **B**): If the ligand-receptors act in a co-operative method, then invasion by EBA-175 (Route 1) and RH5 (Route 2) are not independent of each other. Thus, in the presence of anti-EBA-175_RII_ and anti-rRH5 antibodies, the expected inhibition from the combination anti-sera is only as effective as the most active individual antibody. ***Antibody Steric Hindrance*** (Panels **C** and **D**): Data suggests that EBA-175 abundance is greater than RH5 and is possibly released before RH5 (Ord et al, unpublished observations). At LOW antibody concentrations (**C**), there is no possible hindrance of RH5 by EBA-175, and all available antibodies are able to bind to their respective ligands independently. This is observed as growth inhibition with the combination anti-sera being more effective than that observed with the individual antibodies, i.e., it follows the Independent Antibody Interaction Model (**A**). Conversely, at HIGH antibody concentrations (**D**), anti-EBA-175 antibodies are able to bind to available EBA-175 ligands but they sterically hinder some RH5 antibody/ligand interactions, leaving some RH5 ligands available for invasion through the RH5 ligand/receptor pathways. This is observed as growth inhibition with the combination anti-sera being only as effective as anti-EBA-175_RII_ sera alone, i.e. it follows the Co-Operative Antibody Interaction Model (**B**).

The most interesting outcome of this study was the dual effect seen with the hybrid EBA-175_RII_/rRH5 immunogen at either end of the concentration spectrum. The lower concentrations of antibody yielded the desirable “synergy effect” with the combination antigen inhibiting parasite invasion more efficiently than the individual components (additive effect). This effect dissipates at ∼100 µg/ml and transitions to an inhibition profile that resembles a profile where each antibody seems to act independently (by 250 and 500 µg/ml), and the inhibition we obtain parallels that of the most active component. To understand this dual effect we have come up with a model that explains the outcomes based on whether the ligands (EBA-175 and RH5) act independently of each other or co-operatively. It would appear that when antibody is present at lower concentrations, the lack of steric hindrance allows the antibodies to exert an additive inhibitory effect (Figures 4 and 7). However, as the antibody concentration rises, the antibody against EBA-175 (the EBA-175 ligand is present in higher amounts than RH5 and also comes into contact with the red cell earlier (Ord et al, unpublished observations) saturates the surface of the erythrocyte not allowing anti-RH5 to mediate an effective inhibitory response (Figure 7). Thus, at higher concentrations of the combo antibody what is apparent in the IIA is only the inhibitory effect of anti-EBA-175_RII_ IgG. This steric hindrance model may explain why the synergy seen with the hybrid immunogen at low concentrations disappears mid-range.

From the human vaccine point of view, this data is quite encouraging, as realistic titers from current human trials [34] indicate that they would approach the lower end of the IIA concentration spectrum used in this study. Thus, in the event of multiple antigen immunizations, synergistic effects among the antibodies to yield potent inhibitory effects on merozoite invasion may actually be the desirable outcome.

## Methods

### Ethics statement

All animal work in this study was carried out at A&G Pharmaceutical, Columbia, MD. The A&G Institutional Animal Care and Use Commitee reviewed and approved the animal protocols (protocol number AG-01) to ensure they met with strict accordance to the recommendations of the Guide for the Care and Use of Laboratory Animals of the NIH, and with accordance to the PHS Policy at A&G Pharmaceutical (OLAW AWA #A4404-01). Isoflurane was used to sedate the mice for immunizations, and all efforts were made to minimize suffering at all times.

### Parasite culture

*P. falciparum* 3D7 and Dd2 lines were obtained from the Malaria Research and Reference Reagent Resource Center (MR4) and cultured in human type A+ erythrocytes as described [40]. The identity of each strain has been confirmed by microsatellite fingerprinting [41].

### Recombinant protein synthesis

Region II of the EBA-175 protein was synthesized by Cambrex Biosciences Inc., as described [34], [42]. Full-length RH5 was produced by the wheat germ cell-free system (CellFree Sciences, Matsuyama, Japan) as described elsewhere [30], [43]. Briefly, the full-length RH5 [comprising amino acid (aa) _26_Glu to aa _526_Gln of the 3D7 sequence without signal peptide, Met at N-terminus, and a hexa-histidine (HIS) tag at C-terminus] was cloned into the wheat germ cell-free expression vector, pEU-E01-MCS (CellFree Sciences) at XhoI/NotI sites. The recombinant RH5 protein, ∼63 kDa with HIS-tag, was expressed using wheat germ cell-free system (CellFree Sciences) and purified using Nickel-Sepharose column (GE Healthcare, Camarillo, CA) as described previously [31].

### Mouse immunizations/antibody production & purification

Balb/c mice in groups of 5, received one primary immunization and three boosts over a period of 2 months with either EBA-175_RII_ alone, rRH5 alone, or an equal combination of both. Precision Antibody Proprietary Technology was used for all the immunizations. Ascites and cardiac bleed sera were obtained from all three regimes and pre-immune sera was collected from non-immunized mice. The IgG fraction from ascites sera were purified using Protein G sepharose beads (GE Healthcare) and dialyzed against 1x PBS overnight.

### Erythrocyte binding assays

The recombinant EBA-175_RII_ and RH5 proteins were incubated with type A+ erythrocytes for 2 hr at room temperature. Erythrocytes were then washed with RPMI 1640 incomplete medium, layered over dibutylsulphate oil (Sigma), and centrifuged at 6,000 x *g* for 1 min. The supernatant and oil were removed by aspiration. Bound parasite proteins were eluted from the erythrocytes with 1.5 M NaCl and the eluate was used for immunoprecipitation/Western Blotting with 10 µg purified anti-EBA-175_RII_ or anti-rRH5 antibodies.

### Immunoprecipitation/Western blotting

Saponin-lysed pellets from mature-stage parasites, or lysate obtained from erythrocyte binding assays, were separated under reducing conditions with either 6% or 10% SDS-PAGE gels, as appropriate. Anti-EBA-175_RII_ or anti-rRH5, along with appropriate secondary antibodies, were used to detect specific immuno-reactivity. Mouse pre-immune sera was used as a control.

### Immunofluorescence Assay (IFAs)

Mature schizont stage 3D7 parasites were smeared onto slides and stored at −70°C. Slides were thawed, fixed with 10% methanol/90% acetone for 20 min at room temperature. After air-drying, the smears were blocked and then incubated at room temperature for 1 hr with serum containing the anti-EBA-175_RII_, anti-rRH5 or anti-EBA-175_RII_/rRH5 antibodies, and then incubated with FITC-conjugated anti-mouse antibody (1∶100) for 1 hr at room temperature protected from light. All slides were mounted using 10 µg/mL DAPI and observed under UV light. Images were merged using Adobe Photoshop Elements 6.0 software.

### Neuraminidase treatment of erythrocytes

Cells were treated with 0.025 U/mL neuraminidase (*Vibrio cholerae*; Roche), at 37°C for 1 hr. The efficacy of each enzyme treatment was assessed in the Laboratory of Immunohematology, New York Blood Center, by assaying for loss of erythrocyte agglutinability, using a panel of monoclonal antibodies against suitable antigenic determinants on different blood group proteins.

### Invasion inhibition assays (IIAs)

Mature trophozoite stages were isolated on 40%/70%/90% Percoll gradients as previously described [44]. Cultures for IIAs were established using a starting parasitaemia of 0.8% for 3D7 and 0.6% for Dd2, at a 5% haematocrit. Purified anti-EBA-175_RII_, anti-rRH5, and combination anti-EBA-175_RII_/anti-rRH5 antibodies were tested independently using 3D7 and IgG dilutions ranging from 1.0 µg/mL to 500 µg/mL. A second combination was also tested: equal proportions of anti-EBA-175_RII_ IgG and anti-rRH5 IgG were combined together after purification and tested at final concentrations of 1.0 µg/mL to 500 µg/mL. Purified IgG from mouse sera (Sigma) was used at equivalent concentrations as negative controls. IIAs with Dd2 were performed at antibody concentrations of 1.0, to 100 µg/mL. IIAs at 100 µg/mL and 250 µg/mL with 3D7 were also performed on erythrocytes that had been treated with neuraminidase. All IIAs were assessed after 24–30 hr by enumerating infected erythrocytes on Giemsa stained smears. All IIAs were performed at least 3 times and the representative results from one such assay is shown in the figure.
